# Hemoptysis in patients of celiac disease with disproportionately severe anemia: tip of the iceberg?

**DOI:** 10.1186/2049-6958-8-25

**Published:** 2013-03-21

**Authors:** Kamal Kumar Singhal, Ashok K Janmeja, Rakhee Sodhi, Rajpal S Punia

**Affiliations:** 1 Department of Pediatrics, Government Medical College and Hospital, Sector-32, Chandigarh 160030, India; 2 Department of Pulmonary, Medicine Government Medical College and Hospital, Sector-32, Chandigarh 160030, India; 3 Department of Pathology, Government Medical College and Hospital, Sector-32, Chandigarh 160030, India

**Keywords:** Ceelen–Gellerstedt, Celiac, GFD, Gluten, Hemoptysis, Hemosiderosis, IPH, Lane Hamilton

## Abstract

Idiopathic Pulmonary Hemosiderosis (IPH) is characterized by the triad of iron deficiency anemia, pulmonary infiltrates and haemoptysis with no recognizable cause. Since the first description of its association with Celiac Disease (CD) by Lane and Hamilton in 1971, only a few isolated cases have been reported in literature. Although it has been considered an uncommon association of two disease entities, recent reports indicate that prevalence of celiac disease is as high as one percent. Further, individually both celiac disease and IPH are known to present as refractory anemia only. We are reporting a young adult with Lane Hamilton Syndrome, who realized that he was having significant gastrointestinal complaints only when they disappeared on gluten free diet (GFD). This case report reiterates the fact that celiac disease should be considered in all patients of IPH because of the therapeutic implications. Further on review of literature, we believe that covert hemoptysis may be responsible for disproportionately severe anemia in patients of celiac disease. Thus, prevalence of this association may be more than currently believed. Further research in this regard may improve our understanding of pathogenesis of celiac disease.

## Background

Idiopathic Pulmonary Hemosiderosis (IPH) is a rare disease found primarily in children that causes recurrent episodes of diffuse alveolar hemorrhage. Recurrent alveolar bleeding may eventually produce pulmonary hemosiderosis and fibrosis. IPH is characterized by hemoptysis with no recognizable cause, dyspnea, alveolar opacities on chest radiographs, and iron deficiency anemia. In 1971 Lane and Hamilton first described the association of IPH with Celiac Disease (CD) and since then a few isolated cases have been reported in literature. While the association of CD with IPH can be considered uncommon, recent reports indicate that prevalence of CD can be as high as one percent. Furthermore, both celiac disease and IPH can present a refractory anemia only. Hereinafter we report on a case of IPH where the association with CD was discovered on the basis of anamnestic and clinical data.

## Case presentation

A 27 year old male, non-smoker and non alcoholic, resident of Punjab, India, presented to the outpatient department complaining cough and hemoptysis for two month. Cough was present off and on with mild specks of blood initially. One day prior to presentation patient had hemoptysis of around 400 ml fresh blood. There was no associated fever, weight loss, or decreased appetite. There was no history of close contact with any tubercular patient. He had taken four drug antitubercular therapy (ATT) for about one month, prior to visiting us but without any improvement. The reason for starting ATT and the exact dose was not clear as the patient did not have any written records.

The patient was a thin man with a marked pale complexion. Patient’s height, weight, and BMI were 165 cm, 52 kg and 19.1, respectively. Blood pressure, heart rate and respiratory rate were 116/76 mmHg, 110 per minute and 20 per minute, respectively. Chest examination revealed occasional crepitations bilaterally. The remaining part of the systemic examination was unremarkable.

Lab investigations showed severe microcytic, hypochromic anemia (Hb = 5 g/dl). Blood total leukocyte count was 8.9 × 10^9^/L with 58% polymorphs 40% lymphocytes and 2% eosinophils. The platelet count was 210 × 10^9^/Liter of blood. Coagulation profile, renal and liver function tests were normal. Urine examination did not reveal albuminuria or hematuria. Sputum examination for Acid Fast Bacilli and Tuberculin skin test were negative. Elisa for HIV was non-reactive. Arterial blood gas analysis revealed pH 7.4, PaO_2_ 82.3 mm Hg, PaCO_2_ 42 mmHg, HCO_3_^-^ 23 mEq/L. Chest radiograph on the day of admission showed bilateral diffuse alveolar infiltrates over middle and lower zones, while X- ray taken one month back was normal. Flexible fiberoptic bronchoscopy revealed normal airways. Bronchoalveolar lavage showed numerous hemosiderin laden macrophages.

**Figure 1:**
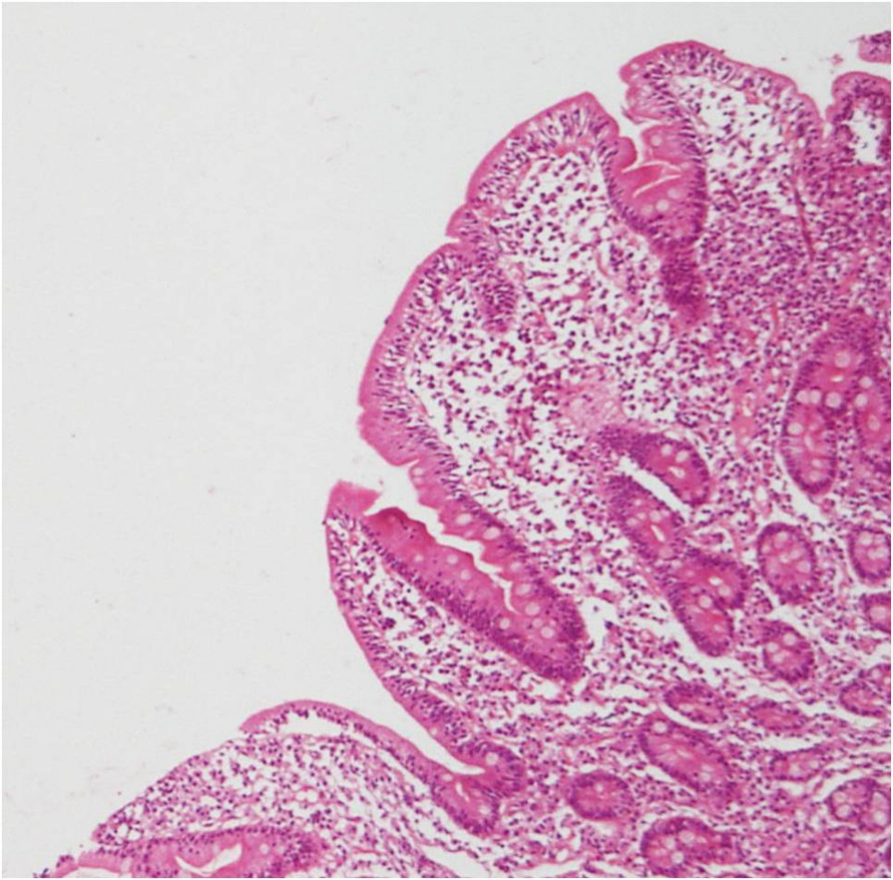
Photomicrograph showing short and broad villi and lymphomononuclear cell infiltrate in lamina propria (H&E x 100).

**Figure 2:**
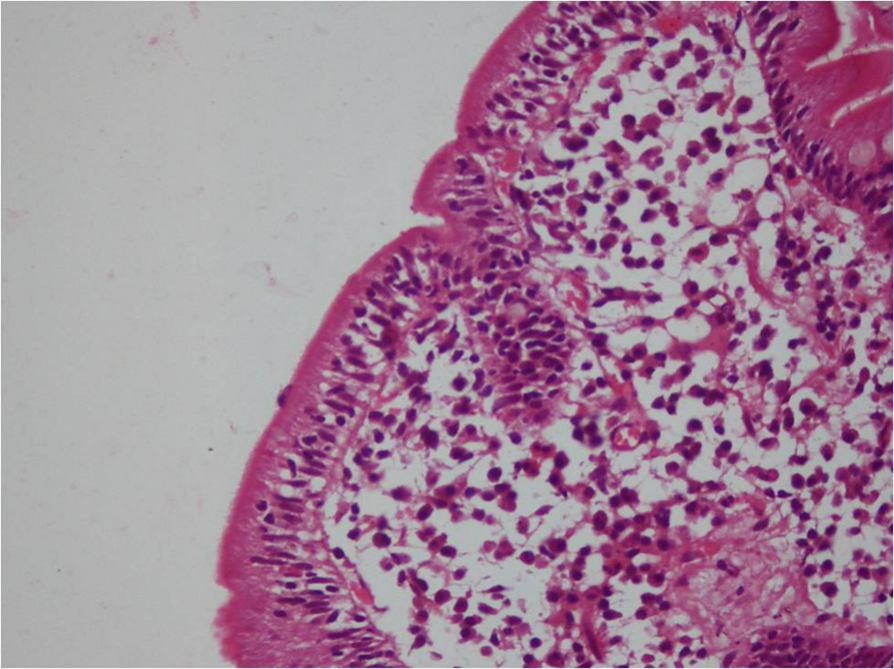
Photomicrograph showing increased intraepithelial lymphocytes (H&E x 200)cb.

Antinuclear, anti-neutrophil cytoplasmic (p-ANCA and c-ANCA), and anti-glomerular basement membrane antibodies were negative. C3 and C4 levels were normal. Echocardiography and electrocardiography provided normal findings. After excluding secondary causes of diffuse alveolar hemorrhage, diagnosis of idiopathic pulmonary hemosiderosis (IPH) was done. Since an association between celiac disease (CD) and IPH has been previously described, a workup for CD was also performed. IgA tissue transglutaminase (TTG) titre was 178 IU/L (normal: ≤10 IU/L). Upper GI endoscopy on gross examination did not reveal any significant abnormality. Duodenal biopsy showed partial villous atrophy, increased numbers of intra-epithelial lymphocytes (70 IELs per 100 epithelial cells), and infiltration of the lamina propria with plasma cells, confirming the diagnosis of celiac disease (Figure 1 and 2). On reviewing the history of the patient, he reported a vague abdominal discomfort after feeds since childhood but never significant enough to warrant any treatment. The discomfort used to subside on its own in 1 to 1.5 hours and he used to pass one formed stool per day.

At admission the patient was given empirical antibiotics and blood transfusion. There was a marked clinical response to blood transfusion. After the diagnosis of associated Celiac Disease, the patient was started on Gluten free diet (GFD). He was discharged after one week. At the time of discharge the patient was asymptomatic and his Chest X-ray revealed resolution of earlier noted shadows. He did not have fever or recurrence of hemoptysis during the hospitalization. Over a follow up period of one year, on GFD, the patient has gained twelve kg weight. There has not been any recurrence of pulmonary symptoms and his hemoglobin level is normal (11.8 g/dl). His abdominal symptoms, which were present since early childhood, also disappeared on GFD. Chest x-ray after one year is normal.

## Discussion

Pulmonary haemorrhage implies bleeding into the lungs and the conducting airways. The term pulmonary hemosiderosis (PH) although often used synonymously with pulmonary hemorrhage is a pathologic diagnosis and denotes the presence of hemosiderin laden macrophages. It is diagnostic of bleeding into the lungs as a result of any etiology. Pulmonary haemorrage may be diffuse or focal. The chest radiograph findings in diffuse alveolar haemorrhage (DAH) are non-specific and consist of an alveolar filling process that can be a patchy, focal, or diffuse alveolar filling process.

Idiopathic pulmonary hemosiderosis (IPH) is a very rare cause of DAH. Diagnosis of IPH requires evidence of DAH and exclusion of other causes of DAH such as cardiac diseases, coagulopathies, connective-tissue disorders, systemic vasculitis, or anti-basement-membrane-antibody diseases. An extensive list of causes of DAH in children and adults is presented in Table 1. Most of the causes can be ruled out clinically but for some of them further investigations are required. DAH is characterized clinically by combination of iron deficiency anemia, pulmonary infiltrates on imaging and hemosiderin-laden macrophages in sputum, gastric aspirate, or bronchoalveolar lavage with or without hemoptysis.

**Table 1:** Causes of diffuse alveolar hemorrhage in children and adults

**Immune mediated**	**Nonimmune mediated**
➣ Idiopathic pulmonary capillaritis	➣ Idiopathic pulmonary hemosiderosis
➣ ANCA associated vasculitis	➣ Acute idiopathic pulmonary hemorrhage of infancy
○ Wegener’s granulomatosis,	➣ Heiner’s syndrome
○ Microscopic polyangiitis,	➣ Asphyxiation/abuse
○ Churg-Strauss syndrome	➣ Cardiovascular causes
➣ Goodpasture’s syndrome	○ Pulmonary vein atresia/stenosis
➣ Systemic lupus erythematosus	○ Total anomalous pulmonary venous return
➣ Henoch-Schönleinpurpura	○ Pulmonary veno-occlusive disease
➣ Behçet’s disease	○ Mitral stenosis
➣ Cryoglobulinemic vasculitis	○ Left-sided heart failure
➣ Juvenile idiopathic arthritis/Rheumatoid arthritis	○ Pulmonary capillary hemangiomatosis
➣ Polymyositis	○ Pulmonary telangiectasia
➣ Mixed connective tissue disease	➣ Neoplasm/hamartomatous
➣ Primary or Secondary antiphospholipid syndrome	○ Angiosarcoma
	○ Lymphangioleiomyomatosis
	○ Tuberous sclerosis
	Severe coagulopathy
	Inhalational toxins
	○ Trimetallic anhydride
	○ Crack cocaine
	Drug Induced pulmonary haemorrhage

This association of IPH and CD was first described by Lane and Hamilton in 1971 [1]. An elaborate review of literature was published by Agarwal in 2007 [2]. In a comprehensive review of literature, we could find twelve additional case reports (Table 2). Thus a total of 35 patients with Lane Hamilton syndrome have been reported in 29 case reports so far. Despite rarity, the conspicuous therapeutic implications make this association important. Out of these thirty five patients, thirteen (37.1%) were adults (above eighteen years of age).

**Table 2:** Summary of reported Lane Hamilton syndromes after the review of literature published by Agarwal*

**S. no.**	**Authors**	**Diseases associated**	**N. of patients reported**	**Improved pulmonary symptoms with GFD**	**Age at diagnosis**	**Abdsympt**	**CD**	
							**Serology positive CD**	**Biopsy proven CD**
1	Paksu S, 2011 [3]	IPH + CD	1	Yes	4.5 yrs	Yes	Yes	Yes
2	Grover PJ, (BJN), 2010 [4]	IPH + CD + epilepsy and cerebral calcification syndrome	1	No	21 yrs	Yes		
3	Hendrickx GF et al., 2011 [5]	IPH + CD	1	Yes	3.5 yr	Yes	Yes	Yes
4	Keskin O et al., 2011 [6]	IPH + CD + Retinitis Pigmentosa	1	NS	9 yr	Yes	Yes	Yes
5	Sethi GR, 2010 [7]	CD + IPH	3	Yes	7–14 yr	No	Yes	Yes
6	Najada AS. Ann Trop Paed, 2010 [8]	CD + IPH	1	No	13 yr	Yes	Yes	Yes
7	Nishino M. Radiology, 2010 [9]	CD + IPH	1	NS	50 yr	Yes	Yes	Yes
8	Narula N, 2010 [10]	CD + IPH + CMP	1	NR	13 yr	No	Yes	Yes
9	Hammami S et al., 2008 [11]	CD + IPH	1	Yes	11 yr	No	Yes	Yes
10	Mayes DH et al., 2008 [12]	CD + IPH	1	Yes	40 yr	No	Yes	Yes
11	Khemiri M et al., 2008 [13]	CD + IPH	3	Yes	1 yr, 5 yr, 11 yr	Yes	Yes	Yes
12	Agarwal R. 2007 [2]	CD + IPH	1	Yes	34 yrs	No	Yes	Yes
13	Hoca NT et al., 2006 [14]	CD + IPH	1	Yes	15 yr	Yes	Yes	Yes

*[2].

AbdSympt, Abdominal symptoms; CD, Celiac disease; CMP, Cardiomyopathy; GFD, Gluten-free diet; IPH, Idiopathic Pulmonary Hemosiderosis; NR, Not reported; NS, Not studied.

Out of the total 35 cases reported till now only 18 (51.4%) had gastrointestinal symptoms. The proportion of patients with gastrointestinal symptoms was greater in adults (53.8%) than in children (50%).

Out of the 35 patients of Lane Hamilton 19(54.2%) demonstrated improvement in pulmonary symptoms with GFD. Pulmonary symptoms responded to GFD in 5/13(38.4%) adults and 14/22(63.6%) children. Thus age seems to be an important factor determining the impact of GFD on pulmonary manifestations, i.e. younger the age group more likely is the response to GFD.

Dramatic improvement in pulmonary symptoms with Gluten free diet was first reported by Reading et al. [15]. The largest series of patients in literature showing response in IPH with GFD has been reported by Khemiri et al. and Sethi et al. [7,13]. Khemiri et al. reported clinical and radiological improvement in both pulmonary and gastrointestinal symptoms with corticosteroid treatment combined with a gluten-free diet. Interestingly, in one of the patients who stopped the gluten-free diet, the symptoms of both IPH and CD recurred. Pacheco et al. reported that GFD induced remission in IPH can be on long-term basis up to 4 years [16]. Sethi et al. reported a patient who was initially followed up as IPH [7]. The pulmonary symptoms persisted despite steroids and azathioprine over a follow up period of two and half year. After discovering associated celiac disease the patient was started on GFD and he did not have recurrence of pulmonary symptoms over a follow up period of 12 months, and it was possible to wean the patient off the glucocorticoid therapy.

Lane-Hamilton syndrome has been considered an association of two different disease entities, with an eluding pathogenetic link between them. We believe that there are good reasons to suggest that Lane Hamilton syndrome may represent two manifestations of a single disease, especially in cases showing response of pulmonary symptoms to GFD. Hemosiderosis in celiac disease is likely to occur due to deposition of immune complexes involving an autoantigen like gluten on alveolar basement membrane, or to direct reaction between the antigen of alveolar basement membrane and an antibody like the antireticulin one. The prevalence of Lane Hamilton disease may not be as rare as it is currently believed. The facts that favour our assumption are as follows:

1. Several studies have suggested a high prevalence of celiac disease (1.8%–14.6%) in patients of Iron Deficiency Anemia (IDA) of obscure origin [17]. This prevalence is as high as 20% in IDA resistant to treatment with iron. Unsworth et al. reported IDA as the only abnormality in 40% of patients of CD [18]. In some of these studies, loss of iron in feces also has been ruled out as the cause of IDA [19,20]. So, there is definite evidence showing association between celiac disease and IDA of obscure origin especially the one not responding to iron therapy.

2. With recurrent alveolar haemorrhage, intra-alveolar and interstitial hemosiderin is deposited in the lungs. This results in Iron-deficiency anemia despite normal total body iron stores, since hemosiderin within alveolar macrophages is not available to the developing erythrocytes.

3. Moreover, even in severe DAH, hemoptysis is not universal, and the diagnosis could be suspected due to progressive anemia only.

Combining these facts it should not be difficult to imagine that a proportion of patients with Lane Hamilton syndrome will be misdiagnosed as celiac only, if there is no overt pulmonary bleed (clinical or radiological).

Therefore a high index of suspicion should be kept regarding pulmonary hemosiderosis in patients of celiac disease with disproportionately severe anemia. And any respiratory illness (e.g. hemoptysis, cough, asthma, pneumonia) associated with aggravation of anemia should prompt a gastroenterologist to actively look for hemosiderosis. This can be safely and noninvasively done by histopathological examination of sputum/induced sputum for hemosiderin laden macrophages.

As in our case, many patients of celiac disease starting GFD realized that many of their gastrointestinal problems which they had perceived as almost normal, disappeared [21]. Since half of the cases with Lane Hamilton syndrome in literature had no bowel involvement, we suggest that all patients of IPH should be investigated for CD.

## Conclusion

In order to define a major therapeutic implication in a disease with otherwise no definitive treatment, it is imperative to investigate every patient of IPH for celiac disease. Similarly, a high index of suspicion for pulmonary hemosiderosis should be kept in patients of celiac disease with disproportionately severe anemia. These patients can be noninvasively investigated for pulmonary hemosiderosis with sputum or induced sputum examination for hemosiderin laden macrophages. Lane Hamilton syndrome may not be rare, but it is rarely reported probably due to a low index of suspicion for celiac disease by pulmonologists and for pulmonary hemosiderosis by gastroenterologists, respectively.

## Consent

Written informed consent was obtained from the patient for publication of this Case report and any accompanying images. A copy of the written consent is available for review by the Editor-in-Chief of this journal.

## Competing interests

The authors declare that they have no competing interest.

## Authors’ contributions

KKS, AKJ, RS and RSP were all involved in conception, design, analysis and interpretation of data, drafting the article, revising it critically for important intellectual content and final approval of the version.

## Funding

This research received no specific funding.
